# Supplementary material for: Potential of Fatty Acids in Treating Sarcopenia: A Systematic Review
Source: Nutrients. 2023 Aug 17;15(16):3613. doi: 10.3390/nu15163613 (PMC10459935; doi:10.3390/nu15163613)
Supplement: Supplementary file 1 [file nutrients-15-03613-s001.zip › supplementary materials-sum.pdf]

Embase

1 fatty acid/ or lipid/ or essential fatty acid/ or hydroxy fatty acid/ or long chain fatty acid/ or medium chain fatty acid/ or short chain fatty acid/ or unsaturated fatty acid/

2 sarcopenia/dt, pc, su, th [Drug Therapy, Prevention, Surgery, Therapy]

1 and 2

Pubmed

1 (fatty acid/ or lipid/ or essential fatty acid/ or hydroxy fatty acid/ or long chain fatty acid/ or medium chain fatty acid/ or short chain fatty acid/ or unsaturated fatty acid/) AND ((sarcopenia) OR (sarcopenic)) (fatty acid/ or lipid/ or essential fatty acid/ or hydroxy fatty acid/ or long chain fatty acid/ or medium chain fatty acid/ or short chain fatty acid/ or unsaturated fatty acid/) AND ((sarcopenia) OR (sarcopenic))

Web of Science

1 (((((((ALL=(fatty acid)) OR ALL=(lipid)) OR ALL=(essential fatty acid)) OR ALL=(hydroxy fatty acid)) OR ALL=(long chain fatty acid)) OR ALL=(medium chain fatty acid)) OR ALL=(short chain fatty acid)) OR ALL=(unsaturated fatty acid
2 ALL=(sarcopenia)
3 ((ALL=(diet therapy)) OR ALL=(drug therapy)) OR ALL=(Therapy)

#3 AND #2 AND #1

Study ID	D1	D2	D3	D4	D5	Overall
Claire. el. (2021)	+	+	+	+	!	!
Emelie el. (2019)	-	+	+	+	!	-
Gordon. el. (2011)	+	+	+	+	!	!
Gordon. el. (2015)	+	+	+	!	!	!
Jun el. (2016)	-	+	+	+	!	-
Mariasole el. (2017)	+	+	+	+	!	!
Mats el. (2020)	+	+	+	-	!	!
Roma el. (2015)	-	+	+	+	!	-
Sebastiaan el. (2021)	+	+	+	+	!	!
Stephan el. (2020)	+	+	+	+	+	+
Stephen el. (2018)	+	+	+	+	+	+
Stephen. el. (2009)	+	+	+	+	!	!
Yves el. (2019)	+	+	+	+	+	+

+ Low risk

! Some concerns

- High risk

D1 Randomisation process

D2 Deviations from the intended interventions

D3 Missing outcome data

D4 Measurement of the outcome

D5 Selection of the reported result

Full text	Abbreviations
B-cell lymphoma 2	BCL2
bcl-2-like protein 4	BAX
Caspase-3	CASP3
Caspase-7	CASP7
Caspase-9	CASP9
Control group	Con
Cytochrome b-c1 complex subunit 1	UQCRC1
Experiment group	Exp
Forkhead box protein M1	FOXM1
Forkhead box protein O1	FOXO1
Glycogen synthase kinase-3 beta	GSK3β
Histone deacetylase 4	HDAC-4
Insulin-like growth factor 2	IGF-II
Insulin-like growth factor-binding protein 5	IGFBP-5
Interleukin 6	IL-6
Interleukin-1 beta	IL-1β
Mammalian target of rapamycin	mTOR
Microtubule-associated proteins 1A/1B light chain 3B	LC3b
Mitochondrial uncoupling protein 3	UCP3
Muscle mass	MM
Muscle performance	MP
Muscle strength	MS
Myosin heavy chain 2	MYH2
Myosin heavy chain 4	MYH4
Myosin heavy chain 7	MYH7
Nuclear receptor co-repressor 1	NCOR1
Post-intervention	Po
Pre-intervention	Pre
Protein kinase B	Akt
RAC(Rho family)-alpha serine/threonine-protein kinase	AKT1
Randomized Controlled Trial	RCT
Tumor necrosis factor-α	TNF-α
Vitamin e	Vit E

Exclusion studies

1. Not primary sarcopenia[1-38]

2. Not using fatty acid as intervention[39-97]

Studies:

1. Kim A, Koo JH, Jin X, Kim W, Park SY, Park S, Rhee EP, Choi CS, Kim SG: **Ablation of USP21 in skeletal muscle promotes oxidative fibre phenotype, inhibiting obesity and type 2 diabetes.** *J Cachexia Sarcopenia Muscle* 2021, **12:**1669-1689.
2. Akashi K, Yokoyama Y, Mizuno T, Abe T, Fukaya M, Asahara T, Nagino M: **Association Between Preoperative Muscle Mass and Intraoperative Bacterial Translocation in Patients Undergoing Hepatectomy, Pancreatoduodenectomy, and Esophagectomy.** *Ann Surg Oncol* 2019, **26:**4805-4813.
3. Catanese S, Beuchel CF, Sawall T, Lordick F, Brauer R, Scholz M, Ceglarek U, Hacker UT: **Biomarkers related to fatty acid oxidative capacity are predictive for continued weight loss in cachectic cancer patients.** *J Cachexia Sarcopenia Muscle* 2021, **12:**2101-2110.
4. Dasarathy S: **Cause and management of muscle wasting in chronic liver disease.** *Current Opinion in Gastroenterology* 2016, **32(3):**159-165.
5. Amor F, Vu Hong A, Corre G, Sanson M, Suel L, Blaie S, Servais L, Voit T, Richard I, Israeli D: **Cholesterol metabolism is a potential therapeutic target in Duchenne muscular dystrophy.** *J Cachexia Sarcopenia Muscle* 2021, **12:**677-693.
6. Dasarathy S: **Consilience in sarcopenia of cirrhosis.** *Journal of Cachexia Sarcopenia and Muscle* 2012, **3:**225-237.
7. Lanchais K, Capel F, Tournadre A: **Could Omega 3 Fatty Acids Preserve Muscle Health in Rheumatoid Arthritis?** *Nutrients* 2020, **12**.
8. Kitada M, Ogura Y, Suzuki T, Monnno I, Kanasaki K, Watanabe A, Koya D: **Cyclic and intermittent very low-protein diet can have beneficial effects against advanced diabetic nephropathy in Wistar fatty (fa/fa) rats, an animal model of type 2 diabetes and obesity.** *Nephrology (Carlton)* 2017, **22:**1030-1034.
9. Lin YL, Liou HH, Lai YH, Wang CH, Kuo CH, Chen SY, Hsu BG: **Decreased serum fatty acid binding protein 4 concentrations are associated with sarcopenia in chronic hemodialysis patients.** *Clin Chim Acta* 2018, **485:**113-118.
10. Halade GV, Rahman MM, Fernandes G: **Differential effects of conjugated linoleic acid isomers in insulin-resistant female C57Bl/6J mice.** *J Nutr Biochem* 2010, **21:**332-337.
11. Tromp J, Voors AA, Sharma A, Ferreira JP, Ouwerkerk W, Hillege HL, Gomez KA, Dickstein K, Anker SD, Metra M, et al: **Distinct Pathological Pathways in Patients With Heart Failure and Diabetes.** *JACC Heart Fail* 2020, **8:**234-242.
12. Deval C, Capel F, Laillet B, Polge C, Béchet D, Taillandier D, Attaix D, Combaret L: **Docosahexaenoic acid-supplementation prior to fasting prevents muscle atrophy in mice.** *J Cachexia Sarcopenia Muscle* 2016, **7:**587-603.
13. Lattanzi B, Giusto M, Albanese C, Mennini G, D'Ambrosio D, Farcomeni A, Ginanni Corradini

S, Rossi M, Merli M: **The Effect of 12 Weeks of β-Hydroxy-β-Methyl-Butyrate Supplementation after Liver Transplantation: A Pilot Randomized Controlled Study.** *Nutrients* 2019, **11**.

14. Harada H, Kai H, Niiyama H, Nishiyama Y, Katoh A, Yoshida N, Fukumoto Y, Ikeda H: **Effectiveness of cardiac rehabilitation for prevention and treatment of sarcopenia in patients with cardiovascular disease - a retrospective cross-sectional analysis.** *Journal of Nutrition, Health and Aging* 2017, **21(4):**449-456.
15. Malafarina V, Uriz-Otano F, Malafarina C, Martinez JA, Zulet MA: **Effectiveness of nutritional supplementation on sarcopenia and recovery in hip fracture patients. A multi-centre randomized trial.** *Maturitas* 2017, **101:**42-50.
16. Teixeira FJ, Matias CN, Monteiro CP, Howell SL, Kones R: **Effects of Alpha-hydroxy-isocaproic acid upon Body Composition in a Type I Diabetic Patient with Muscle Atrophy - A Case Study.** *Yale J Biol Med* 2018, **91:**161-171.
17. Peterson JM, Bryner RW, Frisbee JC, Alway SE: **Effects of exercise and obesity on UCP3 content in rat hindlimb muscles.** *Med Sci Sports Exerc* 2008, **40:**1616-1622.
18. Cauley JA, Manini TM, Lovato L, Talton J, Anton SD, Domanchuk K, Kennedy K, Stowe CL, Walkup M, Fielding RA, et al: **The Enabling Reduction of Low-Grade Inflammation in Seniors (ENRGISE) Pilot Study: Screening Methods and Recruitment Results.** *J Gerontol A Biol Sci Med Sci* 2019, **74:**1296-1302.
19. Marshall K, Winwood RJ: **EPA and DHA Omega 3s as a potential adjunct to chemotherapy in the treatment of cancer.** *Agro Food Industry Hi-Tech* 2017, **28:**19-22.
20. Lee YS, Kim JY, Oh KS, Chung SW: **Fatty acid-binding protein 4 regulates fatty infiltration after rotator cuff tear by hypoxia-inducible factor 1 in mice.** *Journal of Cachexia Sarcopenia and Muscle* 2017, **8:**839-850.
21. Mocellin MC, Camargo CD, Fabre MED, Trindade E: **Fish oil effects on quality of life, body weight and free fat mass change in gastrointestinal cancer patients undergoing chemotherapy: A triple blind, randomized clinical trial.** *Journal of Functional Foods* 2017, **31:**113-122.
22. Mori T, Ohmori H, Luo Y, Mori S, Miyagawa Y, Nukaga S, Goto K, Fujiwara-Tani R, Kishi S, Sasaki T, et al: **Giving combined medium-chain fatty acids and glucose protects against cancer-associated skeletal muscle atrophy.** *Cancer Science* 2019, **110:**3391-3399.
23. Dos Reis AS, Limirio LS, Santos HO, de Oliveira EP: **Intake of polyunsaturated fatty acids and ω-3 are protective factors for sarcopenia in kidney transplant patients.** *Nutrition* 2021, **81:**110929.
24. Lustgarten MS: **The Kidney-Gut-Muscle Axis in End-Stage Renal Disease is Similarly Represented in Older Adults.** *Nutrients* 2019, **12**.
25. Axelrod CL, Fealy CE, Erickson ML, Davuluri G, Fujioka H, Dantas WS, Huang E, Pergola K, Mey JT, King WT, et al: **Lipids activate skeletal muscle mitochondrial fission and quality control networks to induce insulin resistance in humans.** *Metabolism* 2021, **121:**154803.
26. Dillon LM, Hida A, Garcia S, Prolla TA, Moraes CT: **Long-Term Bezafibrate Treatment Improves Skin and Spleen Phenotypes of the mtDNA Mutator Mouse.** *Plos One* 2012, **7**.
27. Tumani MF, Pavez C, Parada A: **Microbiota, dietary habits and diet in inflammatory bowel disease.** *Revista Chilena De Nutricion* 2020, **47:**822-829.
28. Takemura K, Nishi H, Inagi R: **Mitochondrial Dysfunction in Kidney Disease and Uremic**

Sarcopenia. *Frontiers in Physiology* 2020, **11 (no pagination)**.

29. Kumagai H, Coelho AR, Wan J, Mehta HH, Yen K, Huang A, Zempo H, Fuku N, Maeda S, Oliveira PJ, et al: **MOTS-c reduces myostatin and muscle atrophy signaling.** *Am J Physiol Endocrinol Metab* 2021, **320:**E680-e690.
30. Singh SS, Kumar A, Welch N, Sekar J, Mishra S, Bellar A, Gangadhariah M, Attaway A, Al Khafaji H, Wu X, et al: **Multiomics-Identified Intervention to Restore Ethanol-Induced Dysregulated Proteostasis and Secondary Sarcopenia in Alcoholic Liver Disease.** *Cell Physiol Biochem* 2021, **55:**91-116.
31. Laviano A, Di Lazzaro L, Koverech A: **Nutrition support and clinical outcome in advanced cancer patients.** *Proceedings of the Nutrition Society* 2018, **77:**388-393.
32. Noreik M: **Nutritional Therapy for Cachexia and Sarcopenia.** *Aktuelle Ernahrungsmedizin* 2014, **39:**117-126.
33. Okamura T, Hashimoto Y, Miki A, Kaji A, Sakai R, Iwai K, Osaka T, Ushigome E, Hamaguchi M, Yamazaki M, Fukui M: **Reduced dietary omega-3 fatty acids intake is associated with sarcopenia in elderly patients with type 2 diabetes: a cross-sectional study of KAMOGAWA-DM cohort study.** *J Clin Biochem Nutr* 2020, **66:**233-237.
34. Welch AA, Hayhoe RPG, Cameron D: **The relationships between sarcopenic skeletal muscle loss during ageing and macronutrient metabolism, obesity and onset of diabetes.** *Proc Nutr Soc* 2020, **79:**158-169.
35. Glass C, Hipskind P, Tsien C, Malin SK, Kasumov T, Shah SN, Kirwan JP, Dasarathy S: **Sarcopenia and a physiologically low respiratory quotient in patients with cirrhosis: a prospective controlled study.** *Journal of Applied Physiology* 2013, **114:**559-565.
36. Davis MP, Panikkar R: **Sarcopenia associated with chemotherapy and targeted agents for cancer therapy.** *Ann Palliat Med* 2019, **8:**86-101.
37. Dasarathy S, Merli M: **Sarcopenia from mechanism to diagnosis and treatment in liver disease.** *Journal of Hepatology* 2016, **65:**1232-1244.
38. Kappus MR, Mendoza MS, Nguyen D, Medici V, McClave SA: **Sarcopenia in Patients with Chronic Liver Disease: Can It Be Altered by Diet and Exercise?** *Curr Gastroenterol Rep* 2016, **18:**43.
39. Pinzariu AC, Pasca SA, Sindilar A, Drochioi C, Balan M, Oboroceanu T, Niculescu S, Crauciuc DV, Crauciuc EG, Luca A, et al: **Adipose Tissue Remodeling by Prolonged Administration of High Dose of Vitamin D3 in Rats Treated to Prevent Sarcopenia.** *Revista De Chimie* 2017, **68:**2139-2143.
40. Alhussain MH, MM AL: **Association Between Fish Consumption and Muscle Mass and Function in Middle-Age and Older Adults.** *Front Nutr* 2021, **8:**746880.
41. Esmaeily Z, Tajary Z, Daei S, Rezaei M, Eyvazkhani A, Dorosty Motlagh AR, Palmowski A: **Association between Healthy Eating Index-2015 scores and probable sarcopenia in community-dwelling Iranian older adults: a cross-sectional study.** *J Nutr Sci* 2021, **10:**e20.
42. Pereyra AS, Wang ZM, Messi ML, Zhang T, Wu H, Register TC, Forbes E, Devarie-Baez NO, Files DC, Abba MC, et al: **BDA-410 Treatment Reduces Body Weight and Fat Content by Enhancing Lipolysis in Sedentary Senescent Mice.** *J Gerontol A Biol Sci Med Sci* 2017, **72:**1045-1053.
43. Li FH, Sun L, Zhu M, Li T, Gao HE, Wu DS, Zhu L, Duan R, Liu TC: **Beneficial alterations in body composition, physical performance, oxidative stress, inflammatory markers, and**

adipocytokines induced by long-term high-intensity interval training in an aged rat model. *Exp Gerontol* 2018, **113:**150-162.

44. Montiel-Rojas D, Santoro A, Nilsson A, Franceschi C, Capri M, Bazzocchi A, Battista G, de Groot L, Feskens EJM, Berendsen AAM, et al: **Beneficial Role of Replacing Dietary Saturated Fatty Acids with Polyunsaturated Fatty Acids in the Prevention of Sarcopenia: Findings from the NU-AGE Cohort.** *Nutrients* 2020, **12**.
45. Margolis LM, Rivas DA, Ezzyat Y, Gaffney-Stomberg E, Young AJ, McClung JP, Fielding RA, Pasiakos SM: **Calorie Restricted High Protein Diets Downregulate Lipogenesis and Lower Intrahepatic Triglyceride Concentrations in Male Rats.** *Nutrients* 2016, **8**.
46. Vallejo J, Spence M, Cheng AL, Brotto L, Edens NK, Garvey SM, Brotto M: **Cellular and Physiological Effects of Dietary Supplementation with β-Hydroxy-β-Methylbutyrate (HMB) and β-Alanine in Late Middle-Aged Mice.** *PLoS One* 2016, **11:**e0150066.
47. Smith A, Gray J: **Considering the benefits of egg consumption for older people at risk of sarcopenia.** *Br J Community Nurs* 2016, **21:**305-309.
48. Russ DW, Acksel C, Boyd IM, Maynard J, McCorkle KW, Edens NK, Garvey SM: **Dietary HMB and β-alanine co-supplementation does not improve in situ muscle function in sedentary, aged male rats.** *Appl Physiol Nutr Metab* 2015, **40:**1294-1301.
49. Khanal P, He L, Degens H, Stebbings GK, Onambele-Pearson GL, Williams AG, Thomis M, Morse CI: **Dietary Protein Requirement Threshold and Micronutrients Profile in Healthy Older Women Based on Relative Skeletal Muscle Mass.** *Nutrients* 2021, **13**.
50. Din USU, Brook MS, Selby A, Quinlan J, Boereboom C, Abdulla H, Franchi M, Narici MV, Phillips BE, Williams JW, et al: **A double-blind placebo controlled trial into the impacts of HMB supplementation and exercise on free-living muscle protein synthesis, muscle mass and function, in older adults.** *Clin Nutr* 2019, **38:**2071-2078.
51. Stout JR, Smith-Ryan AE, Fukuda DH, Kendall KL, Moon JR, Hoffman JR, Wilson JM, Oliver JS, Mustad VA: **Effect of calcium β-hydroxy-β-methylbutyrate (CaHMB) with and without resistance training in men and women 65+yrs: a randomized, double-blind pilot trial.** *Exp Gerontol* 2013, **48:**1303-1310.
52. Berton L, Bano G, Carraro S, Veronese N, Pizzato S, Bolzetta F, De Rui M, Valmorbida E, De Ronch I, Perissinotto E, et al: **Effect of Oral Beta-Hydroxy-Beta-Methylbutyrate (HMB) Supplementation on Physical Performance in Healthy Old Women Over 65 Years: An Open Label Randomized Controlled Trial.** *PLoS One* 2015, **10:**e0141757.
53. Sacheck JM, Milbury PE, Cannon JG, Roubenoff R, Blumberg JB: **Effect of vitamin E and eccentric exercise on selected biomarkers of oxidative stress in young and elderly men.** *Free Radic Biol Med* 2003, **34:**1575-1588.
54. Deutz NE, Pereira SL, Hays NP, Oliver JS, Edens NK, Evans CM, Wolfe RR: **Effect of β-hydroxy-β-methylbutyrate (HMB) on lean body mass during 10 days of bed rest in older adults.** *Clin Nutr* 2013, **32:**704-712.
55. Bennett BT, Mohamed JS, Alway SE: **The Effects of Calcium-β-Hydroxy-β-Methylbutyrate on Aging-Associated Apoptotic Signaling and Muscle Mass and Function in Unloaded but Nonatrophied Extensor Digitorum Longus Muscles of Aged Rats.** *Oxid Med Cell Longev* 2020, **2020:**3938672.
56. Osuka Y, Kojima N, Sasai H, Wakaba K, Miyauchi D, Tanaka K, Kim H: **Effects of exercise and/or β-hydroxy-β-methylbutyrate supplementation on muscle mass, muscle strength,**

and physical performance in older women with low muscle mass: a randomized, double-blind, placebo-controlled trial. *Am J Clin Nutr* 2021, **114:**1371-1385.

57. Alhussain MH, Alkahtani S, Aljuhani O, Habib SS: **Effects of Nutrient Intake on Diagnostic Measures of Sarcopenia among Arab Men: A Cross-Sectional Study.** *Nutrients* 2020, **13**.
58. Osuka Y, Kojima N, Wakaba K, Miyauchi D, Tanaka K, Kim H: **Effects of resistance training and/or beta-hydroxy-beta-methylbutyrate supplementation on muscle mass, muscle strength and physical performance in older women with reduced muscle mass: protocol for a randomised, double-blind, placebo-controlled trial.** *BMJ Open* 2019, **9:**e025723.
59. Russ DW, Acksel C, McCorkle KW, Edens NK, Garvey SM: **Effects of Running Wheel Activity and Dietary HMB and β-alanine Co-Supplementation on Muscle Quality in Aged Male Rats.** *J Nutr Health Aging* 2017, **21:**554-561.
60. Di Cesare Mannelli L, Micheli L, Lucarini E, Parisio C, Toti A, Tenci B, Zanardelli M, Branca JJV, Pacini A, Ghelardini C: **Effects of the Combination of β-Hydroxy-β-Methyl Butyrate and R(+) Lipoic Acid in a Cellular Model of Sarcopenia.** *Molecules* 2020, **25**.
61. Standley RA, Distefano G, Pereira SL, Tian M, Kelly OJ, Coen PM, Deutz NEP, Wolfe RR, Goodpaster BH: **Effects of β-hydroxy-β-methylbutyrate on skeletal muscle mitochondrial content and dynamics, and lipids after 10 days of bed rest in older adults.** *J Appl Physiol (1985)* 2017, **123:**1092-1100.
62. Colica C, Merra G, Gasbarrini A, De Lorenzo A, Cioccoloni G, Gualtieri P, Perrone MA, Bernardini S, Bernardo V, Di Renzo L, Marchetti M: **Efficacy and safety of very-low-calorie ketogenic diet: a double blind randomized crossover study.** *European Review for Medical and Pharmacological Sciences* 2017, **21:**2274-2289.
63. Chow SK, Chim YN, Cheng KY, Ho CY, Ho WT, Cheng KC, Wong RM, Cheung WH: **Elastic-band resistance exercise or vibration treatment in combination with hydroxymethylbutyrate (HMB) supplement for management of sarcopenia in older people: a study protocol for a single-blinded randomised controlled trial in Hong Kong.** *BMJ Open* 2020, **10:**e034921.
64. Bidon C, Lachuer J, Molgó J, Wierinckx A, de la Porte S, Pignol B, Christen Y, Meloni R, Koenig H, Biguet NF, Mallet J: **The extract of Ginkgo biloba EGb 761 reactivates a juvenile profile in the skeletal muscle of sarcopenic rats by transcriptional reprogramming.** *PLoS One* 2009, **4:**e7998.
65. Bidon C, Lachuer J, Molgo J, Wierinckx A, de la Porte S, Pignol B, Christen Y, Meloni R, Koenig H, Faucon-Biguet N, Mallet J: **The Extract of Ginkgo biloba EGb 761 Reactivates a Juvenile Profile in the Skeletal Muscle of Sarcopenic Rats by Transcriptional Reprogramming.** *Plos One* 2009, **4**.
66. Corsetto PA, Montorfano G, Klersy C, Massimino L, Infantino V, Iannello G, Anna Faliva M, Lukaski H, Perna S, Alalwan TA, et al: **Fatty Acid Profile and Antioxidant Status Fingerprint in Sarcopenic Elderly Patients: Role of Diet and Exercise.** *Nutrients* 2019, **11**.
67. Stern RA, Mozdziak PE: **Glutamine synthetase in avian muscle contributes to a positive myogenic response to ammonia compared with mammalian muscle.** *Am J Physiol Regul Integr Comp Physiol* 2019, **317:**R214-r221.
68. Kim JH, Kim JH, Jang JP, Jang JH, Jin DH, Kim YS, Jin HJ: **Identification of Molecules from Coffee Silverskin That Suppresses Myostatin Activity and Improves Muscle Mass and Strength in Mice.** *Molecules* 2021, **26**.

69. Ahn J, Kim MJ, Yoo A, Ahn J, Ha TY, Jung CH, Seo HD, Jang YJ: **Identifying Codium fragile extract components and their effects on muscle weight and exercise endurance.** *Food Chem* 2021, **353:**129463.
70. Konopka AR, Laurin JL, Musci RV, Wolff CA, Reid JJ, Biela LM, Zhang Q, Peelor FF, 3rd, Melby CL, Hamilton KL, Miller BF: **Influence of Nrf2 activators on subcellular skeletal muscle protein and DNA synthesis rates after 6 weeks of milk protein feeding in older adults.** *Geroscience* 2017, **39:**175-186.
71. Lee CC, Liao YC, Lee MC, Lin KJ, Hsu HY, Chiou SY, Young SL, Lin JS, Huang CC, Watanabe K: **Lactobacillus plantarum TWK10 Attenuates Aging-Associated Muscle Weakness, Bone Loss, and Cognitive Impairment by Modulating the Gut Microbiome in Mice.** *Front Nutr* 2021, **8:**708096.
72. Baptista IL, Silva WJ, Artioli GG, Guilherme JP, Leal ML, Aoki MS, Miyabara EH, Moriscot AS: **Leucine and HMB differentially modulate proteasome system in skeletal muscle under different sarcopenic conditions.** *PLoS One* 2013, **8:**e76752.
73. Park S, Yuan H, Zhang T, Wu X, Huang SK, Cho SM: **Long-term silk peptide intake promotes skeletal muscle mass, reduces inflammation, and modulates gut microbiota in middle-aged female rats.** *Biomedicine and Pharmacotherapy* 2021, **137 (no pagination)**.
74. Jang IY, Jung HW, Park JH, Kim JH, Lee S, Lee E, Lee JY, Park SJ, Kim DA, Kim SJ, et al: **Lower Serum n-3 Fatty Acid Level in Older Adults with Sarcopenia.** *Nutrients* 2020, **12**.
75. Jyväkorpi SK, Urtamo A, Kivimäki M, Strandberg TE: **Macronutrient composition and sarcopenia in the oldest-old men: The Helsinki Businessmen Study (HBS).** *Clin Nutr* 2020, **39:**3839-3841.
76. Nasimi N, Sohrabi Z, Dabbaghmanesh MH, Eskandari MH, Bedeltavana A, Famouri M, Talezadeh P: **A Novel Fortified Dairy Product and Sarcopenia Measures in Sarcopenic Older Adults: A Double-Blind Randomized Controlled Trial.** *Journal of the American Medical Directors Association* 2021, **22(4):**809-815.
77. Peng LN, Cheng YC, Yu PC, Lee WJ, Lin MH, Chen LK: **Oral Nutritional Supplement with β-hydroxy-β-methylbutyrate (HMB) Improves Nutrition, Physical Performance and Ameliorates Intramuscular Adiposity in Pre-Frail Older Adults: A Randomized Controlled Trial.** *J Nutr Health Aging* 2021, **25:**767-773.
78. Ma SL, Wu J, Zhu L, Chan RS, Wang X, Huang D, Tang NL, Woo J: **Peripheral Blood T Cell Gene Expression Responses to Exercise and HMB in Sarcopenia.** *Nutrients* 2021, **13**.
79. Zanchi NE, Guimaraes-Ferreira L, Siqueira MA, Camporez JPG, Nicastro H, Chaves DFS, Campos-Ferraz P, Lancha AH, Carvalho CRD: **The possible role of leucine in modulating glucose homeostasis under distinct catabolic conditions.** *Medical Hypotheses* 2012, **79:**883-888.
80. Rossi AP, D'Introno A, Rubele S, Caliari C, Gattazzo S, Zoico E, Mazzali G, Fantin F, Zamboni M: **The Potential of β-Hydroxy-β-Methylbutyrate as a New Strategy for the Management of Sarcopenia and Sarcopenic Obesity.** *Drugs Aging* 2017, **34:**833-840.
81. Chung E, Mo HB, Wang S, Zu YJ, Elfakhani M, Rios SR, Chyu MC, Yang RS, Shen CL: **Potential roles of vitamin E in age-related changes in skeletal muscle health.** *Nutrition Research* 2018, **49:**23-36.
82. Standley RA, Liu SZ, Jemiolo B, Trappe SW, Trappe TA: **Prostaglandin E2 induces transcription of skeletal muscle mass regulators interleukin-6 and muscle RING finger-1**

in humans. *Prostaglandins Leukot Essent Fatty Acids* 2013, **88:**361-364.

83. Funakoshi T, Kanzaki N, Otsuka Y, Izumo T, Shibata H, Machida S: **Quercetin inhibits adipogenesis of muscle progenitor cells in vitro.** *Biochem Biophys Rep* 2018, **13:**39-44.

84. Villani A, Wright H, Slater G, Buckley J: **A randomised controlled intervention study investigating the efficacy of carotenoid-rich fruits and vegetables and extra-virgin olive oil on attenuating sarcopenic symptomology in overweight and obese older adults during energy intake restriction: protocol paper.** *BMC Geriatr* 2018, **18:**2.

85. Huang Y, Zhu X, Chen K, Lang H, Zhang Y, Hou P, Ran L, Zhou M, Zheng J, Yi L, et al: **Resveratrol prevents sarcopenic obesity by reversing mitochondrial dysfunction and oxidative stress via the PKA/LKB1/AMPK pathway.** *Aging (Albany NY)* 2019, **11:**2217-2240.

86. Sanz-Paris A, Camprubi-Robles M, Lopez-Pedrosa JM, Pereira SL, Rueda R, Ballesteros-Pomar MD, Garcia Almeida JM, Cruz-Jentoft AJ: **Role of Oral Nutritional Supplements Enriched with β-Hydroxy-β-Methylbutyrate in Maintaining Muscle Function and Improving Clinical Outcomes in Various Clinical Settings.** *J Nutr Health Aging* 2018, **22:**664-675.

87. Niu K, Guo H, Guo Y, Ebihara S, Asada M, Ohrui T, Furukawa K, Ichinose M, Yanai K, Kudo Y, et al: **Royal jelly prevents the progression of sarcopenia in aged mice in vivo and in vitro.** *J Gerontol A Biol Sci Med Sci* 2013, **68:**1482-1492.

88. Kim TN, Won JC, Kim YJ, Lee EJ, Kim MK, Park MS, Lee SK, Kim JM, Ko KS, Rhee BD: **Serum adipocyte fatty acid-binding protein levels are independently associated with sarcopenic obesity.** *Diabetes Res Clin Pract* 2013, **101:**210-217.

89. Rivas DA, Rice NP, Ezzyat Y, McDonald DJ, Cooper BE, Fielding RA: **Sphingosine-1-phosphate analog FTY720 reverses obesity but not age-induced anabolic resistance to muscle contraction.** *American Journal of Physiology-Cell Physiology* 2019, **317:**C502-C512.

90. Malafarina V, Uriz-Otano F, Gil-Guerrero L, Iniesta R, Zulet MA, Martinez JA: **Study protocol: High-protein nutritional intervention based on β-hydroxy-β-methylbutirate, vitamin D3 and calcium on obese and lean aged patients with hip fractures and sarcopenia. The HIPERPROT-GER study.** *Maturitas* 2013, **76:**123-128.

91. Nakano H, Hashimoto H, Mochizuki M, Naraba H, Takahashi Y, Sonoo T, Matsuishi Y, Ogawa Y, Shimojo N, Inoue Y, Nakamura K: **Urinary Titin N-Fragment Evaluation in a Randomized Controlled Trial of Beta-Hydroxy-Beta-Methylbutyrate for Acute Mild Trauma in Older Adults.** *Nutrients* 2021, **13**.

92. Wang J, Cui C, Chim YN, Yao H, Shi L, Xu J, Wang J, Wong RMY, Leung KS, Chow SK, Cheung WH: **Vibration and β-hydroxy-β-methylbutyrate treatment suppresses intramuscular fat infiltration and adipogenic differentiation in sarcopenic mice.** *J Cachexia Sarcopenia Muscle* 2020, **11:**564-577.

93. Pannerec A, Migliavacca E, De Castro A, Michaud J, Karaz S, Goulet L, Rezzi S, Ng TP, Bosco N, Larbi A, Feige JN: **Vitamin B12 deficiency and impaired expression of amnionless during aging.** *Journal of Cachexia Sarcopenia and Muscle* 2018, **9:**41-52.

94. Molmen KS, Hammarstrom D, Pedersen K, Lian Lie AC, Steile RB, Nygaard H, Khan Y, Hamarsland H, Koll L, Hanestadhaugen M, et al: **Vitamin D<inf>3</inf> supplementation does not enhance the effects of resistance training in older adults.** *Journal of Cachexia, Sarcopenia and Muscle* 2021, **12(3):**599-628.

95. Alway SE, Pereira SL, Edens NK, Hao Y, Bennett BT: **β-Hydroxy-β-methylbutyrate (HMB)**

enhances the proliferation of satellite cells in fast muscles of aged rats during recovery from disuse atrophy. *Exp Gerontol* 2013, **48:**973-984.

96. Hao Y, Jackson JR, Wang Y, Edens N, Pereira SL, Alway SE: **β-Hydroxy-β-methylbutyrate reduces myonuclear apoptosis during recovery from hind limb suspension-induced muscle fiber atrophy in aged rats.** *Am J Physiol Regul Integr Comp Physiol* 2011, **301:**R701-715.

97. Ahn J, Son HJ, Seo HD, Ha TY, Ahn J, Lee H, Shin SH, Jung CH, Jang YJ: **γ-Oryzanol Improves Exercise Endurance and Muscle Strength by Upregulating PPARδ and ERRγ Activity in Aged Mice.** *Mol Nutr Food Res* 2021, **65:**e2000652.

AMSTAR 2: a critical appraisal tool for systematic reviews that include randomised or non-randomised studies of healthcare interventions, or both

1. Did the research questions and inclusion criteria for the review include the components of PICO?

For Yes:
- [x] Population
- [x] Intervention
- [x] Comparator group
- [x] Outcome

Optional (recommended)
- [ ] Timeframe for follow-up

- [x] Yes
- [ ] No

2. Did the report of the review contain an explicit statement that the review methods were established prior to the conduct of the review and did the report justify any significant deviations from the protocol?

For Partial Yes:
The authors state that they had a written protocol or guide that included ALL the following:
- [x] review question(s)
- [x] a search strategy
- [x] inclusion/exclusion criteria
- [x] a risk of bias assessment

For Yes:
As for partial yes, plus the protocol should be registered and should also have specified:
- [ ] a meta-analysis/synthesis plan, if appropriate, *and*
- [ ] a plan for investigating causes of heterogeneity
- [ ] justification for any deviations from the protocol

- [ ] Yes
- [x] Partial Yes
- [ ] No

3. Did the review authors explain their selection of the study designs for inclusion in the review?

For Yes, the review should satisfy ONE of the following:
- [x] *Explanation for* including only RCTs
- [ ] OR *Explanation for* including only NRSI
- [ ] OR *Explanation for* including both RCTs and NRSI

- [x] Yes
- [ ] No

4. Did the review authors use a comprehensive literature search strategy?

For Partial Yes (all the following):
- [x] searched at least 2 databases (relevant to research question)
- [x] provided key word and/or search strategy
- [x] justified publication restrictions (e.g. language)

For Yes, should also have (all the following):
- [ ] searched the reference lists / bibliographies of included studies
- [ ] searched trial/study registries
- [ ] included/consulted content experts in the field
- [ ] where relevant, searched for grey literature
- [ ] conducted search within 24 months of completion of the review

- [ ] Yes
- [x] Partial Yes
- [ ] No

5. Did the review authors perform study selection in duplicate?

For Yes, either ONE of the following:
- [x] at least two reviewers independently agreed on selection of eligible studies and achieved consensus on which studies to include
- [ ] OR two reviewers selected a sample of eligible studies and achieved good agreement (at least 80 percent), with the remainder selected by one reviewer.

- [x] Yes
- [ ] No

6. Did the review authors perform data extraction in duplicate?

For Yes, either ONE of the following:

- [x] at least two reviewers achieved consensus on which data to extract from included studies
- [ ] OR two reviewers extracted data from a sample of eligible studies and achieved good agreement (at least 80 percent), with the remainder extracted by one reviewer.

- [x] Yes
- [ ] No

7. Did the review authors provide a list of excluded studies and justify the exclusions?

For Partial Yes:

- [ ] provided a list of all potentially relevant studies that were read in full-text form but excluded from the review

For Yes, must also have:

- [x] Justified the exclusion from the review of each potentially relevant study

- [x] Yes
- [ ] Partial Yes
- [ ] No

8. Did the review authors describe the included studies in adequate detail?

For Partial Yes (ALL the following):

- [x] described populations
- [x] described interventions
- [x] described comparators
- [x] described outcomes
- [ ] described research designs

For Yes, should also have ALL the following:

- [ ] described population in detail
- [ ] described intervention in detail (including doses where relevant)
- [ ] described comparator in detail (including doses where relevant)
- [ ] described study's setting
- [ ] timeframe for follow-up

- [ ] Yes
- [x] Partial Yes
- [ ] No

9. Did the review authors use a satisfactory technique for assessing the risk of bias (RoB) in individual studies that were included in the review?

RCTs

For Partial Yes, must have assessed RoB from

- [x] unconcealed allocation, *and*
- [x] lack of blinding of patients and assessors when assessing outcomes (unnecessary for objective outcomes such as all-cause mortality)

For Yes, must also have assessed RoB from:

- [ ] allocation sequence that was not truly random, *and*
- [ ] selection of the reported result from among multiple measurements or analyses of a specified outcome

- [x] Yes
- [ ] Partial Yes
- [ ] No
- [ ] Includes only NRSI

NRSI

For Partial Yes, must have assessed RoB:

- [ ] from confounding, *and*
- [ ] from selection bias

For Yes, must also have assessed RoB:

- [ ] methods used to ascertain exposures and outcomes, *and*
- [ ] selection of the reported result from among multiple measurements or analyses of a specified outcome

- [ ] Yes
- [ ] Partial Yes
- [x] No
- [ ] Includes only RCTs

10. Did the review authors report on the sources of funding for the studies included in the review?

For Yes

- [ ] Must have reported on the sources of funding for individual studies included in the review. Note: Reporting that the reviewers looked for this information but it was not reported by study authors also qualifies

- [ ] Yes
- [x] No

11. If meta-analysis was performed did the review authors use appropriate methods for statistical combination of results?

RCTs

For Yes:

- ☐ The authors justified combining the data in a meta-analysis
  - ☐ AND they used an appropriate weighted technique to combine study results and adjusted for heterogeneity if present.
  - ☐ AND investigated the causes of any heterogeneity

- ☐ Yes
- ☐ No
- ☑ No meta-analysis conducted

For NRSI

For Yes:

- ☐ The authors justified combining the data in a meta-analysis
  - ☐ AND they used an appropriate weighted technique to combine study results, adjusting for heterogeneity if present
  - ☐ AND they statistically combined effect estimates from NRSI that were adjusted for confounding, rather than combining raw data, or justified combining raw data when adjusted effect estimates were not available
  - ☐ AND they reported separate summary estimates for RCTs and NRSI separately when both were included in the review

- ☐ Yes
- ☐ No
- ☑ No meta-analysis conducted

12. If meta-analysis was performed, did the review authors assess the potential impact of RoB in individual studies on the results of the meta-analysis or other evidence synthesis?

For Yes:

- ☐ included only low risk of bias RCTs
- ☐ OR, if the pooled estimate was based on RCTs and/or NRSI at variable RoB, the authors performed analyses to investigate possible impact of RoB on summary estimates of effect.

- ☐ Yes
- ☐ No
- ☑ No meta-analysis conducted

13. Did the review authors account for RoB in individual studies when interpreting/ discussing the results of the review?

For Yes:

- ☐ included only low risk of bias RCTs
- ☑ OR, if RCTs with moderate or high RoB, or NRSI were included the review provided a discussion of the likely impact of RoB on the results

- ☑ Yes
- ☐ No

14. Did the review authors provide a satisfactory explanation for, and discussion of, any heterogeneity observed in the results of the review?

For Yes:

- ☐ There was no significant heterogeneity in the results
- ☑ OR if heterogeneity was present the authors performed an investigation of sources of any heterogeneity in the results and discussed the impact of this on the results of the review

- ☑ Yes
- ☐ No

15. If they performed quantitative synthesis did the review authors carry out an adequate investigation of publication bias (small study bias) and discuss its likely impact on the results of the review?

For Yes:

- ☐ performed graphical or statistical tests for publication bias and discussed the likelihood and magnitude of impact of publication bias

- ☐ Yes
- ☐ No
- ☑ No meta-analysis conducted

16. Did the review authors report any potential sources of conflict of interest, including any funding they received for conducting the review?

For Yes:<br>☑ The authors reported no competing interests OR<br>☐ The authors described their funding sources and how they managed potential conflicts of interest	☑ Yes<br>☐ No

To cite this tool: Shea BJ, Reeves BC, Wells G, Thuku M, Hamel C, Moran J, Moher D, Tugwell P, Welch V, Kristjansson E, Henry DA. AMSTAR 2: a critical appraisal tool for systematic reviews that include randomised or non-randomised studies of healthcare interventions, or both. BMJ. 2017 Sep 21;358:j4008.

RCTs				Jadad Quality Assessment scale
**Number**	**study**	**Experimental design**	**supplementation**	**Quality assessment**
1	Roma el. (2015)	RCT	n-3 PUFA+ Vit E<br>Vit E (Con)	3 （Medium）
2	Mariasole el. (2017)	RCT	n–3 PUFA<br>placebo (Con)<br>All with lower-limb resistance exercise training	4 （Good）
3	Emelie el. (2019)	RCT	resistance training and healthy diet rich in N-3PUFAs (RT-HD),<br>resistance training only (RT)<br>controls (CON)	3 (Medium)
4	Stephen el. (2018)	RCT	resistance training and omega-3 (Exp)<br>placebo (Con)	5 (High)
5	Sebastiaan el. (2021)	RCT	Resistance exercise and Omega-3 (Exp)<br>corn oil (Con)	4 (Good)
6	Stephan el. (2020)	double-blind, RCT	1) 2000 IU vitamin D-3 per day;<br>2) 4000 mg CLA per day;<br>3) both Vit D (2000 IU/d) and CLA (4000 mg/d); 4) placebo	5 (High)
7	Yves el. (2019)	multi center, RCT	1) ω3-PUFA alone,<br>2) ω3-PUFA plus the multidomain intervention 3) the multidomain intervention plus placebo,<br>4) placebo alone.	5 (High)

8	Jun el. (2016)	double-blind RCT	PUFA<br>corn oil (Con)	4 (Good)
9	Claire. el. (2021)	single center, parallel, double-blind, RCT	Electrical Muscle Stimulation Procedures+<br>1)placebo capsules<br>2)Whey protein isolate capsules<br>3)WPI+Omega-4+rutin, and curcumin	4 (Good)
10	Gordon. el. (2015)	double-blind RCT	Corn oil (CON)<br>n–3 PUFA group	3 (Medium)
11	Gordon. el. (2011)	RCT	omega-3 fatty acids (Exp)<br>corn oil(Con)	4 (Good)
12	Stephen. el. (2009)	double-blind, RCT	alpha-linolenic acid (ALA)<br>placebo (Con)	4 (Good)
13	Mats el. (2020)	double-blind, RCT	Resistance exercise and omega-3 (Exp)<br>placebo (Con)	5 (High)
