# Supplementary material for: Potential of Fatty Acids in Treating Sarcopenia: A Systematic Review
Source: Nutrients. 2023 Aug 17;15(16):3613. doi: 10.3390/nu15163613 (PMC10459935; doi:10.3390/nu15163613)
Supplement: Supplementary file 1 [file nutrients-15-03613-s001.zip › supplementary materials1-search strategy.pdf]

Embase

1 fatty acid/ or lipid/ or essential fatty acid/ or hydroxy fatty acid/ or long chain fatty acid/ or medium chain fatty acid/ or short chain fatty acid/ or unsaturated fatty acid/

2 sarcopenia/dt, pc, su, th [Drug Therapy, Prevention, Surgery, Therapy]

1 and 2

Pubmed

1 (fatty acid/ or lipid/ or essential fatty acid/ or hydroxy fatty acid/ or long chain fatty acid/ or medium chain fatty acid/ or short chain fatty acid/ or unsaturated fatty acid/) AND ((sarcopenia) OR (sarcopenic)) (fatty acid/ or lipid/ or essential fatty acid/ or hydroxy fatty acid/ or long chain fatty acid/ or medium chain fatty acid/ or short chain fatty acid/ or unsaturated fatty acid/) AND ((sarcopenia) OR (sarcopenic))

Web of Science

1 (((((((ALL=(fatty acid)) OR ALL=(lipid)) OR ALL=(essential fatty acid)) OR ALL=(hydroxy fatty acid)) OR ALL=(long chain fatty acid)) OR ALL=(medium chain fatty acid)) OR ALL=(short chain fatty acid)) OR ALL=(unsaturated fatty acid
2 ALL=(sarcopenia)
3 ((ALL=(diet therapy)) OR ALL=(drug therapy)) OR ALL=(Therapy)

#3 AND #2 AND #1
