# Supplementary material for: Potential of Fatty Acids in Treating Sarcopenia: A Systematic Review
Source: Nutrients. 2023 Aug 17;15(16):3613. doi: 10.3390/nu15163613 (PMC10459935; doi:10.3390/nu15163613)
Supplement: Supplementary file 1 [file nutrients-15-03613-s001.zip › supplementary materials2-Rob2.pdf]

Study ID	D1	D2	D3	D4	D5	Overall
Claire. el. (2021)	+	+	+	+	!	!
Emelie el. (2019)	-	+	+	+	!	-
Gordon. el. (2011)	+	+	+	+	!	!
Gordon. el. (2015)	+	+	+	!	!	!
Jun el. (2016)	-	+	+	+	!	-
Mariasole el. (2017)	+	+	+	+	!	!
Mats el. (2020)	+	+	+	-	!	!
Roma el. (2015)	-	+	+	+	!	-
Sebastiaan el. (2021)	+	+	+	+	!	!
Stephan el. (2020)	+	+	+	+	+	+
Stephen el. (2018)	+	+	+	+	+	+
Stephen. el. (2009)	+	+	+	+	!	!
Yves el. (2019)	+	+	+	+	+	+

+ Low risk

! Some concerns

- High risk

D1 Randomisation process

D2 Deviations from the intended interventions

D3 Missing outcome data

D4 Measurement of the outcome

D5 Selection of the reported result
