# Supplementary material for: Potential of Fatty Acids in Treating Sarcopenia: A Systematic Review
Source: Nutrients. 2023 Aug 17;15(16):3613. doi: 10.3390/nu15163613 (PMC10459935; doi:10.3390/nu15163613)
Supplement: Supplementary file 1 [file nutrients-15-03613-s001.zip › supplementary materials3-abbreviations.pdf]

Full text	Abbreviations
B-cell lymphoma 2	BCL2
bcl-2-like protein 4	BAX
Caspase-3	CASP3
Caspase-7	CASP7
Caspase-9	CASP9
Control group	Con
Cytochrome b-c1 complex subunit 1	UQCRC1
Experiment group	Exp
Forkhead box protein M1	FOXM1
Forkhead box protein O1	FOXO1
Glycogen synthase kinase-3 beta	GSK3β
Histone deacetylase 4	HDAC-4
Insulin-like growth factor 2	IGF-II
Insulin-like growth factor-binding protein 5	IGFBP-5
Interleukin 6	IL-6
Interleukin-1 beta	IL-1β
Mammalian target of rapamycin	mTOR
Microtubule-associated proteins 1A/1B light chain 3B	LC3b
Mitochondrial uncoupling protein 3	UCP3
Muscle mass	MM
Muscle performance	MP
Muscle strength	MS
Myosin heavy chain 2	MYH2
Myosin heavy chain 4	MYH4
Myosin heavy chain 7	MYH7
Nuclear receptor co-repressor 1	NCOR1
Post-intervention	Po
Pre-intervention	Pre
Protein kinase B	Akt
RAC(Rho family)-alpha serine/threonine-protein kinase	AKT1
Randomized Controlled Trial	RCT
Tumor necrosis factor-α	TNF-α
Vitamin e	Vit E
