# Supplementary material for: Potential of Fatty Acids in Treating Sarcopenia: A Systematic Review
Source: Nutrients. 2023 Aug 17;15(16):3613. doi: 10.3390/nu15163613 (PMC10459935; doi:10.3390/nu15163613)
Supplement: Supplementary file 1 [file nutrients-15-03613-s001.zip › supplementary materials4-List of exclusive studies .pdf]

Exclusion studies

1. Not primary sarcopenia[1-38]

2. Not using fatty acid as intervention[39-97]

Studies:

1. Kim A, Koo JH, Jin X, Kim W, Park SY, Park S, Rhee EP, Choi CS, Kim SG: **Ablation of USP21 in skeletal muscle promotes oxidative fibre phenotype, inhibiting obesity and type 2 diabetes.** *J Cachexia Sarcopenia Muscle* 2021, **12:**1669-1689.
2. Akashi K, Yokoyama Y, Mizuno T, Abe T, Fukaya M, Asahara T, Nagino M: **Association Between Preoperative Muscle Mass and Intraoperative Bacterial Translocation in Patients Undergoing Hepatectomy, Pancreatoduodenectomy, and Esophagectomy.** *Ann Surg Oncol* 2019, **26:**4805-4813.
3. Catanese S, Beuchel CF, Sawall T, Lordick F, Brauer R, Scholz M, Ceglarek U, Hacker UT: **Biomarkers related to fatty acid oxidative capacity are predictive for continued weight loss in cachectic cancer patients.** *J Cachexia Sarcopenia Muscle* 2021, **12:**2101-2110.
4. Dasarathy S: **Cause and management of muscle wasting in chronic liver disease.** *Current Opinion in Gastroenterology* 2016, **32(3):**159-165.
5. Amor F, Vu Hong A, Corre G, Sanson M, Suel L, Blaie S, Servais L, Voit T, Richard I, Israeli D: **Cholesterol metabolism is a potential therapeutic target in Duchenne muscular dystrophy.** *J Cachexia Sarcopenia Muscle* 2021, **12:**677-693.
6. Dasarathy S: **Consilience in sarcopenia of cirrhosis.** *Journal of Cachexia Sarcopenia and Muscle* 2012, **3:**225-237.
7. Lanchais K, Capel F, Tournadre A: **Could Omega 3 Fatty Acids Preserve Muscle Health in Rheumatoid Arthritis?** *Nutrients* 2020, **12**.
8. Kitada M, Ogura Y, Suzuki T, Monnno I, Kanasaki K, Watanabe A, Koya D: **Cyclic and intermittent very low-protein diet can have beneficial effects against advanced diabetic nephropathy in Wistar fatty (fa/fa) rats, an animal model of type 2 diabetes and obesity.** *Nephrology (Carlton)* 2017, **22:**1030-1034.
9. Lin YL, Liou HH, Lai YH, Wang CH, Kuo CH, Chen SY, Hsu BG: **Decreased serum fatty acid binding protein 4 concentrations are associated with sarcopenia in chronic hemodialysis patients.** *Clin Chim Acta* 2018, **485:**113-118.
10. Halade GV, Rahman MM, Fernandes G: **Differential effects of conjugated linoleic acid isomers in insulin-resistant female C57Bl/6J mice.** *J Nutr Biochem* 2010, **21:**332-337.
11. Tromp J, Voors AA, Sharma A, Ferreira JP, Ouwerkerk W, Hillege HL, Gomez KA, Dickstein K, Anker SD, Metra M, et al: **Distinct Pathological Pathways in Patients With Heart Failure and Diabetes.** *JACC Heart Fail* 2020, **8:**234-242.
12. Deval C, Capel F, Laillet B, Polge C, Béchet D, Taillandier D, Attaix D, Combaret L: **Docosahexaenoic acid-supplementation prior to fasting prevents muscle atrophy in mice.** *J Cachexia Sarcopenia Muscle* 2016, **7:**587-603.
13. Lattanzi B, Giusto M, Albanese C, Mennini G, D'Ambrosio D, Farcomeni A, Ginanni Corradini

S, Rossi M, Merli M: **The Effect of 12 Weeks of β-Hydroxy-β-Methyl-Butyrate Supplementation after Liver Transplantation: A Pilot Randomized Controlled Study.** *Nutrients* 2019, **11**.

14. Harada H, Kai H, Niiyama H, Nishiyama Y, Katoh A, Yoshida N, Fukumoto Y, Ikeda H: **Effectiveness of cardiac rehabilitation for prevention and treatment of sarcopenia in patients with cardiovascular disease - a retrospective cross-sectional analysis.** *Journal of Nutrition, Health and Aging* 2017, **21(4):**449-456.
15. Malafarina V, Uriz-Otano F, Malafarina C, Martinez JA, Zulet MA: **Effectiveness of nutritional supplementation on sarcopenia and recovery in hip fracture patients. A multi-centre randomized trial.** *Maturitas* 2017, **101:**42-50.
16. Teixeira FJ, Matias CN, Monteiro CP, Howell SL, Kones R: **Effects of Alpha-hydroxy-isocaproic acid upon Body Composition in a Type I Diabetic Patient with Muscle Atrophy - A Case Study.** *Yale J Biol Med* 2018, **91:**161-171.
17. Peterson JM, Bryner RW, Frisbee JC, Alway SE: **Effects of exercise and obesity on UCP3 content in rat hindlimb muscles.** *Med Sci Sports Exerc* 2008, **40:**1616-1622.
18. Cauley JA, Manini TM, Lovato L, Talton J, Anton SD, Domanchuk K, Kennedy K, Stowe CL, Walkup M, Fielding RA, et al: **The Enabling Reduction of Low-Grade Inflammation in Seniors (ENRGISE) Pilot Study: Screening Methods and Recruitment Results.** *J Gerontol A Biol Sci Med Sci* 2019, **74:**1296-1302.
19. Marshall K, Winwood RJ: **EPA and DHA Omega 3s as a potential adjunct to chemotherapy in the treatment of cancer.** *Agro Food Industry Hi-Tech* 2017, **28:**19-22.
20. Lee YS, Kim JY, Oh KS, Chung SW: **Fatty acid-binding protein 4 regulates fatty infiltration after rotator cuff tear by hypoxia-inducible factor 1 in mice.** *Journal of Cachexia Sarcopenia and Muscle* 2017, **8:**839-850.
21. Mocellin MC, Camargo CD, Fabre MED, Trindade E: **Fish oil effects on quality of life, body weight and free fat mass change in gastrointestinal cancer patients undergoing chemotherapy: A triple blind, randomized clinical trial.** *Journal of Functional Foods* 2017, **31:**113-122.
22. Mori T, Ohmori H, Luo Y, Mori S, Miyagawa Y, Nukaga S, Goto K, Fujiwara-Tani R, Kishi S, Sasaki T, et al: **Giving combined medium-chain fatty acids and glucose protects against cancer-associated skeletal muscle atrophy.** *Cancer Science* 2019, **110:**3391-3399.
23. Dos Reis AS, Limirio LS, Santos HO, de Oliveira EP: **Intake of polyunsaturated fatty acids and ω-3 are protective factors for sarcopenia in kidney transplant patients.** *Nutrition* 2021, **81:**110929.
24. Lustgarten MS: **The Kidney-Gut-Muscle Axis in End-Stage Renal Disease is Similarly Represented in Older Adults.** *Nutrients* 2019, **12**.
25. Axelrod CL, Fealy CE, Erickson ML, Davuluri G, Fujioka H, Dantas WS, Huang E, Pergola K, Mey JT, King WT, et al: **Lipids activate skeletal muscle mitochondrial fission and quality control networks to induce insulin resistance in humans.** *Metabolism* 2021, **121:**154803.
26. Dillon LM, Hida A, Garcia S, Prolla TA, Moraes CT: **Long-Term Bezafibrate Treatment Improves Skin and Spleen Phenotypes of the mtDNA Mutator Mouse.** *Plos One* 2012, **7**.
27. Tumani MF, Pavez C, Parada A: **Microbiota, dietary habits and diet in inflammatory bowel disease.** *Revista Chilena De Nutricion* 2020, **47:**822-829.
28. Takemura K, Nishi H, Inagi R: **Mitochondrial Dysfunction in Kidney Disease and Uremic**

Sarcopenia. *Frontiers in Physiology* 2020, **11 (no pagination)**.

29. Kumagai H, Coelho AR, Wan J, Mehta HH, Yen K, Huang A, Zempo H, Fuku N, Maeda S, Oliveira PJ, et al: **MOTS-c reduces myostatin and muscle atrophy signaling.** *Am J Physiol Endocrinol Metab* 2021, **320:**E680-e690.
30. Singh SS, Kumar A, Welch N, Sekar J, Mishra S, Bellar A, Gangadhariah M, Attaway A, Al Khafaji H, Wu X, et al: **Multiomics-Identified Intervention to Restore Ethanol-Induced Dysregulated Proteostasis and Secondary Sarcopenia in Alcoholic Liver Disease.** *Cell Physiol Biochem* 2021, **55:**91-116.
31. Laviano A, Di Lazzaro L, Koverech A: **Nutrition support and clinical outcome in advanced cancer patients.** *Proceedings of the Nutrition Society* 2018, **77:**388-393.
32. Noreik M: **Nutritional Therapy for Cachexia and Sarcopenia.** *Aktuelle Ernahrungsmedizin* 2014, **39:**117-126.
33. Okamura T, Hashimoto Y, Miki A, Kaji A, Sakai R, Iwai K, Osaka T, Ushigome E, Hamaguchi M, Yamazaki M, Fukui M: **Reduced dietary omega-3 fatty acids intake is associated with sarcopenia in elderly patients with type 2 diabetes: a cross-sectional study of KAMOGAWA-DM cohort study.** *J Clin Biochem Nutr* 2020, **66:**233-237.
34. Welch AA, Hayhoe RPG, Cameron D: **The relationships between sarcopenic skeletal muscle loss during ageing and macronutrient metabolism, obesity and onset of diabetes.** *Proc Nutr Soc* 2020, **79:**158-169.
35. Glass C, Hipskind P, Tsien C, Malin SK, Kasumov T, Shah SN, Kirwan JP, Dasarathy S: **Sarcopenia and a physiologically low respiratory quotient in patients with cirrhosis: a prospective controlled study.** *Journal of Applied Physiology* 2013, **114:**559-565.
36. Davis MP, Panikkar R: **Sarcopenia associated with chemotherapy and targeted agents for cancer therapy.** *Ann Palliat Med* 2019, **8:**86-101.
37. Dasarathy S, Merli M: **Sarcopenia from mechanism to diagnosis and treatment in liver disease.** *Journal of Hepatology* 2016, **65:**1232-1244.
38. Kappus MR, Mendoza MS, Nguyen D, Medici V, McClave SA: **Sarcopenia in Patients with Chronic Liver Disease: Can It Be Altered by Diet and Exercise?** *Curr Gastroenterol Rep* 2016, **18:**43.
39. Pinzariu AC, Pasca SA, Sindilar A, Drochioi C, Balan M, Oboroceanu T, Niculescu S, Crauciuc DV, Crauciuc EG, Luca A, et al: **Adipose Tissue Remodeling by Prolonged Administration of High Dose of Vitamin D3 in Rats Treated to Prevent Sarcopenia.** *Revista De Chimie* 2017, **68:**2139-2143.
40. Alhussain MH, MM AL: **Association Between Fish Consumption and Muscle Mass and Function in Middle-Age and Older Adults.** *Front Nutr* 2021, **8:**746880.
41. Esmaeily Z, Tajary Z, Daei S, Rezaei M, Eyvazkhani A, Dorosty Motlagh AR, Palmowski A: **Association between Healthy Eating Index-2015 scores and probable sarcopenia in community-dwelling Iranian older adults: a cross-sectional study.** *J Nutr Sci* 2021, **10:**e20.
42. Pereyra AS, Wang ZM, Messi ML, Zhang T, Wu H, Register TC, Forbes E, Devarie-Baez NO, Files DC, Abba MC, et al: **BDA-410 Treatment Reduces Body Weight and Fat Content by Enhancing Lipolysis in Sedentary Senescent Mice.** *J Gerontol A Biol Sci Med Sci* 2017, **72:**1045-1053.
43. Li FH, Sun L, Zhu M, Li T, Gao HE, Wu DS, Zhu L, Duan R, Liu TC: **Beneficial alterations in body composition, physical performance, oxidative stress, inflammatory markers, and**

adipocytokines induced by long-term high-intensity interval training in an aged rat model. *Exp Gerontol* 2018, **113:**150-162.

44. Montiel-Rojas D, Santoro A, Nilsson A, Franceschi C, Capri M, Bazzocchi A, Battista G, de Groot L, Feskens EJM, Berendsen AAM, et al: **Beneficial Role of Replacing Dietary Saturated Fatty Acids with Polyunsaturated Fatty Acids in the Prevention of Sarcopenia: Findings from the NU-AGE Cohort.** *Nutrients* 2020, **12**.
45. Margolis LM, Rivas DA, Ezzyat Y, Gaffney-Stomberg E, Young AJ, McClung JP, Fielding RA, Pasiakos SM: **Calorie Restricted High Protein Diets Downregulate Lipogenesis and Lower Intrahepatic Triglyceride Concentrations in Male Rats.** *Nutrients* 2016, **8**.
46. Vallejo J, Spence M, Cheng AL, Brotto L, Edens NK, Garvey SM, Brotto M: **Cellular and Physiological Effects of Dietary Supplementation with β-Hydroxy-β-Methylbutyrate (HMB) and β-Alanine in Late Middle-Aged Mice.** *PLoS One* 2016, **11:**e0150066.
47. Smith A, Gray J: **Considering the benefits of egg consumption for older people at risk of sarcopenia.** *Br J Community Nurs* 2016, **21:**305-309.
48. Russ DW, Acksel C, Boyd IM, Maynard J, McCorkle KW, Edens NK, Garvey SM: **Dietary HMB and β-alanine co-supplementation does not improve in situ muscle function in sedentary, aged male rats.** *Appl Physiol Nutr Metab* 2015, **40:**1294-1301.
49. Khanal P, He L, Degens H, Stebbings GK, Onambele-Pearson GL, Williams AG, Thomis M, Morse CI: **Dietary Protein Requirement Threshold and Micronutrients Profile in Healthy Older Women Based on Relative Skeletal Muscle Mass.** *Nutrients* 2021, **13**.
50. Din USU, Brook MS, Selby A, Quinlan J, Boereboom C, Abdulla H, Franchi M, Narici MV, Phillips BE, Williams JW, et al: **A double-blind placebo controlled trial into the impacts of HMB supplementation and exercise on free-living muscle protein synthesis, muscle mass and function, in older adults.** *Clin Nutr* 2019, **38:**2071-2078.
51. Stout JR, Smith-Ryan AE, Fukuda DH, Kendall KL, Moon JR, Hoffman JR, Wilson JM, Oliver JS, Mustad VA: **Effect of calcium β-hydroxy-β-methylbutyrate (CaHMB) with and without resistance training in men and women 65+yrs: a randomized, double-blind pilot trial.** *Exp Gerontol* 2013, **48:**1303-1310.
52. Berton L, Bano G, Carraro S, Veronese N, Pizzato S, Bolzetta F, De Rui M, Valmorbida E, De Ronch I, Perissinotto E, et al: **Effect of Oral Beta-Hydroxy-Beta-Methylbutyrate (HMB) Supplementation on Physical Performance in Healthy Old Women Over 65 Years: An Open Label Randomized Controlled Trial.** *PLoS One* 2015, **10:**e0141757.
53. Sacheck JM, Milbury PE, Cannon JG, Roubenoff R, Blumberg JB: **Effect of vitamin E and eccentric exercise on selected biomarkers of oxidative stress in young and elderly men.** *Free Radic Biol Med* 2003, **34:**1575-1588.
54. Deutz NE, Pereira SL, Hays NP, Oliver JS, Edens NK, Evans CM, Wolfe RR: **Effect of β-hydroxy-β-methylbutyrate (HMB) on lean body mass during 10 days of bed rest in older adults.** *Clin Nutr* 2013, **32:**704-712.
55. Bennett BT, Mohamed JS, Alway SE: **The Effects of Calcium-β-Hydroxy-β-Methylbutyrate on Aging-Associated Apoptotic Signaling and Muscle Mass and Function in Unloaded but Nonatrophied Extensor Digitorum Longus Muscles of Aged Rats.** *Oxid Med Cell Longev* 2020, **2020:**3938672.
56. Osuka Y, Kojima N, Sasai H, Wakaba K, Miyauchi D, Tanaka K, Kim H: **Effects of exercise and/or β-hydroxy-β-methylbutyrate supplementation on muscle mass, muscle strength,**

and physical performance in older women with low muscle mass: a randomized, double-blind, placebo-controlled trial. *Am J Clin Nutr* 2021, **114:**1371-1385.

57. Alhussain MH, Alkahtani S, Aljuhani O, Habib SS: **Effects of Nutrient Intake on Diagnostic Measures of Sarcopenia among Arab Men: A Cross-Sectional Study.** *Nutrients* 2020, **13**.
58. Osuka Y, Kojima N, Wakaba K, Miyauchi D, Tanaka K, Kim H: **Effects of resistance training and/or beta-hydroxy-beta-methylbutyrate supplementation on muscle mass, muscle strength and physical performance in older women with reduced muscle mass: protocol for a randomised, double-blind, placebo-controlled trial.** *BMJ Open* 2019, **9:**e025723.
59. Russ DW, Acksel C, McCorkle KW, Edens NK, Garvey SM: **Effects of Running Wheel Activity and Dietary HMB and β-alanine Co-Supplementation on Muscle Quality in Aged Male Rats.** *J Nutr Health Aging* 2017, **21:**554-561.
60. Di Cesare Mannelli L, Micheli L, Lucarini E, Parisio C, Toti A, Tenci B, Zanardelli M, Branca JJV, Pacini A, Ghelardini C: **Effects of the Combination of β-Hydroxy-β-Methyl Butyrate and R(+) Lipoic Acid in a Cellular Model of Sarcopenia.** *Molecules* 2020, **25**.
61. Standley RA, Distefano G, Pereira SL, Tian M, Kelly OJ, Coen PM, Deutz NEP, Wolfe RR, Goodpaster BH: **Effects of β-hydroxy-β-methylbutyrate on skeletal muscle mitochondrial content and dynamics, and lipids after 10 days of bed rest in older adults.** *J Appl Physiol (1985)* 2017, **123:**1092-1100.
62. Colica C, Merra G, Gasbarrini A, De Lorenzo A, Cioccoloni G, Gualtieri P, Perrone MA, Bernardini S, Bernardo V, Di Renzo L, Marchetti M: **Efficacy and safety of very-low-calorie ketogenic diet: a double blind randomized crossover study.** *European Review for Medical and Pharmacological Sciences* 2017, **21:**2274-2289.
63. Chow SK, Chim YN, Cheng KY, Ho CY, Ho WT, Cheng KC, Wong RM, Cheung WH: **Elastic-band resistance exercise or vibration treatment in combination with hydroxymethylbutyrate (HMB) supplement for management of sarcopenia in older people: a study protocol for a single-blinded randomised controlled trial in Hong Kong.** *BMJ Open* 2020, **10:**e034921.
64. Bidon C, Lachuer J, Molgó J, Wierinckx A, de la Porte S, Pignol B, Christen Y, Meloni R, Koenig H, Biguet NF, Mallet J: **The extract of Ginkgo biloba EGb 761 reactivates a juvenile profile in the skeletal muscle of sarcopenic rats by transcriptional reprogramming.** *PLoS One* 2009, **4:**e7998.
65. Bidon C, Lachuer J, Molgo J, Wierinckx A, de la Porte S, Pignol B, Christen Y, Meloni R, Koenig H, Faucon-Biguet N, Mallet J: **The Extract of Ginkgo biloba EGb 761 Reactivates a Juvenile Profile in the Skeletal Muscle of Sarcopenic Rats by Transcriptional Reprogramming.** *Plos One* 2009, **4**.
66. Corsetto PA, Montorfano G, Klersy C, Massimino L, Infantino V, Iannello G, Anna Faliva M, Lukaski H, Perna S, Alalwan TA, et al: **Fatty Acid Profile and Antioxidant Status Fingerprint in Sarcopenic Elderly Patients: Role of Diet and Exercise.** *Nutrients* 2019, **11**.
67. Stern RA, Mozdziak PE: **Glutamine synthetase in avian muscle contributes to a positive myogenic response to ammonia compared with mammalian muscle.** *Am J Physiol Regul Integr Comp Physiol* 2019, **317:**R214-r221.
68. Kim JH, Kim JH, Jang JP, Jang JH, Jin DH, Kim YS, Jin HJ: **Identification of Molecules from Coffee Silverskin That Suppresses Myostatin Activity and Improves Muscle Mass and Strength in Mice.** *Molecules* 2021, **26**.

69. Ahn J, Kim MJ, Yoo A, Ahn J, Ha TY, Jung CH, Seo HD, Jang YJ: **Identifying Codium fragile extract components and their effects on muscle weight and exercise endurance.** *Food Chem* 2021, **353:**129463.
70. Konopka AR, Laurin JL, Musci RV, Wolff CA, Reid JJ, Biela LM, Zhang Q, Peelor FF, 3rd, Melby CL, Hamilton KL, Miller BF: **Influence of Nrf2 activators on subcellular skeletal muscle protein and DNA synthesis rates after 6 weeks of milk protein feeding in older adults.** *Geroscience* 2017, **39:**175-186.
71. Lee CC, Liao YC, Lee MC, Lin KJ, Hsu HY, Chiou SY, Young SL, Lin JS, Huang CC, Watanabe K: **Lactobacillus plantarum TWK10 Attenuates Aging-Associated Muscle Weakness, Bone Loss, and Cognitive Impairment by Modulating the Gut Microbiome in Mice.** *Front Nutr* 2021, **8:**708096.
72. Baptista IL, Silva WJ, Artioli GG, Guilherme JP, Leal ML, Aoki MS, Miyabara EH, Moriscot AS: **Leucine and HMB differentially modulate proteasome system in skeletal muscle under different sarcopenic conditions.** *PLoS One* 2013, **8:**e76752.
73. Park S, Yuan H, Zhang T, Wu X, Huang SK, Cho SM: **Long-term silk peptide intake promotes skeletal muscle mass, reduces inflammation, and modulates gut microbiota in middle-aged female rats.** *Biomedicine and Pharmacotherapy* 2021, **137 (no pagination)**.
74. Jang IY, Jung HW, Park JH, Kim JH, Lee S, Lee E, Lee JY, Park SJ, Kim DA, Kim SJ, et al: **Lower Serum n-3 Fatty Acid Level in Older Adults with Sarcopenia.** *Nutrients* 2020, **12**.
75. Jyväkorpi SK, Urtamo A, Kivimäki M, Strandberg TE: **Macronutrient composition and sarcopenia in the oldest-old men: The Helsinki Businessmen Study (HBS).** *Clin Nutr* 2020, **39:**3839-3841.
76. Nasimi N, Sohrabi Z, Dabbaghmanesh MH, Eskandari MH, Bedeltavana A, Famouri M, Talezadeh P: **A Novel Fortified Dairy Product and Sarcopenia Measures in Sarcopenic Older Adults: A Double-Blind Randomized Controlled Trial.** *Journal of the American Medical Directors Association* 2021, **22(4):**809-815.
77. Peng LN, Cheng YC, Yu PC, Lee WJ, Lin MH, Chen LK: **Oral Nutritional Supplement with β-hydroxy-β-methylbutyrate (HMB) Improves Nutrition, Physical Performance and Ameliorates Intramuscular Adiposity in Pre-Frail Older Adults: A Randomized Controlled Trial.** *J Nutr Health Aging* 2021, **25:**767-773.
78. Ma SL, Wu J, Zhu L, Chan RS, Wang X, Huang D, Tang NL, Woo J: **Peripheral Blood T Cell Gene Expression Responses to Exercise and HMB in Sarcopenia.** *Nutrients* 2021, **13**.
79. Zanchi NE, Guimaraes-Ferreira L, Siqueira MA, Camporez JPG, Nicastro H, Chaves DFS, Campos-Ferraz P, Lancha AH, Carvalho CRD: **The possible role of leucine in modulating glucose homeostasis under distinct catabolic conditions.** *Medical Hypotheses* 2012, **79:**883-888.
80. Rossi AP, D'Introno A, Rubele S, Caliari C, Gattazzo S, Zoico E, Mazzali G, Fantin F, Zamboni M: **The Potential of β-Hydroxy-β-Methylbutyrate as a New Strategy for the Management of Sarcopenia and Sarcopenic Obesity.** *Drugs Aging* 2017, **34:**833-840.
81. Chung E, Mo HB, Wang S, Zu YJ, Elfakhani M, Rios SR, Chyu MC, Yang RS, Shen CL: **Potential roles of vitamin E in age-related changes in skeletal muscle health.** *Nutrition Research* 2018, **49:**23-36.
82. Standley RA, Liu SZ, Jemiolo B, Trappe SW, Trappe TA: **Prostaglandin E2 induces transcription of skeletal muscle mass regulators interleukin-6 and muscle RING finger-1**

in humans. *Prostaglandins Leukot Essent Fatty Acids* 2013, **88:**361-364.

83. Funakoshi T, Kanzaki N, Otsuka Y, Izumo T, Shibata H, Machida S: **Quercetin inhibits adipogenesis of muscle progenitor cells in vitro.** *Biochem Biophys Rep* 2018, **13:**39-44.

84. Villani A, Wright H, Slater G, Buckley J: **A randomised controlled intervention study investigating the efficacy of carotenoid-rich fruits and vegetables and extra-virgin olive oil on attenuating sarcopenic symptomology in overweight and obese older adults during energy intake restriction: protocol paper.** *BMC Geriatr* 2018, **18:**2.

85. Huang Y, Zhu X, Chen K, Lang H, Zhang Y, Hou P, Ran L, Zhou M, Zheng J, Yi L, et al: **Resveratrol prevents sarcopenic obesity by reversing mitochondrial dysfunction and oxidative stress via the PKA/LKB1/AMPK pathway.** *Aging (Albany NY)* 2019, **11:**2217-2240.

86. Sanz-Paris A, Camprubi-Robles M, Lopez-Pedrosa JM, Pereira SL, Rueda R, Ballesteros-Pomar MD, Garcia Almeida JM, Cruz-Jentoft AJ: **Role of Oral Nutritional Supplements Enriched with β-Hydroxy-β-Methylbutyrate in Maintaining Muscle Function and Improving Clinical Outcomes in Various Clinical Settings.** *J Nutr Health Aging* 2018, **22:**664-675.

87. Niu K, Guo H, Guo Y, Ebihara S, Asada M, Ohrui T, Furukawa K, Ichinose M, Yanai K, Kudo Y, et al: **Royal jelly prevents the progression of sarcopenia in aged mice in vivo and in vitro.** *J Gerontol A Biol Sci Med Sci* 2013, **68:**1482-1492.

88. Kim TN, Won JC, Kim YJ, Lee EJ, Kim MK, Park MS, Lee SK, Kim JM, Ko KS, Rhee BD: **Serum adipocyte fatty acid-binding protein levels are independently associated with sarcopenic obesity.** *Diabetes Res Clin Pract* 2013, **101:**210-217.

89. Rivas DA, Rice NP, Ezzyat Y, McDonald DJ, Cooper BE, Fielding RA: **Sphingosine-1-phosphate analog FTY720 reverses obesity but not age-induced anabolic resistance to muscle contraction.** *American Journal of Physiology-Cell Physiology* 2019, **317:**C502-C512.

90. Malafarina V, Uriz-Otano F, Gil-Guerrero L, Iniesta R, Zulet MA, Martinez JA: **Study protocol: High-protein nutritional intervention based on β-hydroxy-β-methylbutirate, vitamin D3 and calcium on obese and lean aged patients with hip fractures and sarcopenia. The HIPERPROT-GER study.** *Maturitas* 2013, **76:**123-128.

91. Nakano H, Hashimoto H, Mochizuki M, Naraba H, Takahashi Y, Sonoo T, Matsuishi Y, Ogawa Y, Shimojo N, Inoue Y, Nakamura K: **Urinary Titin N-Fragment Evaluation in a Randomized Controlled Trial of Beta-Hydroxy-Beta-Methylbutyrate for Acute Mild Trauma in Older Adults.** *Nutrients* 2021, **13**.

92. Wang J, Cui C, Chim YN, Yao H, Shi L, Xu J, Wang J, Wong RMY, Leung KS, Chow SK, Cheung WH: **Vibration and β-hydroxy-β-methylbutyrate treatment suppresses intramuscular fat infiltration and adipogenic differentiation in sarcopenic mice.** *J Cachexia Sarcopenia Muscle* 2020, **11:**564-577.

93. Pannerec A, Migliavacca E, De Castro A, Michaud J, Karaz S, Goulet L, Rezzi S, Ng TP, Bosco N, Larbi A, Feige JN: **Vitamin B12 deficiency and impaired expression of amnionless during aging.** *Journal of Cachexia Sarcopenia and Muscle* 2018, **9:**41-52.

94. Molmen KS, Hammarstrom D, Pedersen K, Lian Lie AC, Steile RB, Nygaard H, Khan Y, Hamarsland H, Koll L, Hanestadhaugen M, et al: **Vitamin D<inf>3</inf> supplementation does not enhance the effects of resistance training in older adults.** *Journal of Cachexia, Sarcopenia and Muscle* 2021, **12(3):**599-628.

95. Alway SE, Pereira SL, Edens NK, Hao Y, Bennett BT: **β-Hydroxy-β-methylbutyrate (HMB)**

enhances the proliferation of satellite cells in fast muscles of aged rats during recovery from disuse atrophy. *Exp Gerontol* 2013, **48:**973-984.

96. Hao Y, Jackson JR, Wang Y, Edens N, Pereira SL, Alway SE: **β-Hydroxy-β-methylbutyrate reduces myonuclear apoptosis during recovery from hind limb suspension-induced muscle fiber atrophy in aged rats.** *Am J Physiol Regul Integr Comp Physiol* 2011, **301:**R701-715.

97. Ahn J, Son HJ, Seo HD, Ha TY, Ahn J, Lee H, Shin SH, Jung CH, Jang YJ: **γ-Oryzanol Improves Exercise Endurance and Muscle Strength by Upregulating PPARδ and ERRγ Activity in Aged Mice.** *Mol Nutr Food Res* 2021, **65:**e2000652.
