# Supplementary material for: Potential of Fatty Acids in Treating Sarcopenia: A Systematic Review
Source: Nutrients. 2023 Aug 17;15(16):3613. doi: 10.3390/nu15163613 (PMC10459935; doi:10.3390/nu15163613)
Supplement: Supplementary file 1 [file nutrients-15-03613-s001.zip › supplementary materials6-Jadad Quality Assessment.pdf]

RCTs				Jadad Quality Assessment scale
**Number**	**study**	**Experimental design**	**supplementation**	**Quality assessment**
1	Roma el. (2015)	RCT	n-3 PUFA+ Vit E<br>Vit E (Con)	3 （Medium）
2	Mariasole el. (2017)	RCT	n–3 PUFA<br>placebo (Con)<br>All with lower-limb resistance exercise training	4 （Good）
3	Emelie el. (2019)	RCT	resistance training and healthy diet rich in N-3PUFAs (RT-HD),<br>resistance training only (RT)<br>controls (CON)	3 (Medium)
4	Stephen el. (2018)	RCT	resistance training and omega-3 (Exp)<br>placebo (Con)	5 (High)
5	Sebastiaan el. (2021)	RCT	Resistance exercise and Omega-3 (Exp)<br>corn oil (Con)	4 (Good)
6	Stephan el. (2020)	double-blind, RCT	1) 2000 IU vitamin D-3 per day;<br>2) 4000 mg CLA per day;<br>3) both Vit D (2000 IU/d) and CLA (4000 mg/d); 4) placebo	5 (High)
7	Yves el. (2019)	multi center, RCT	1) ω3-PUFA alone,<br>2) ω3-PUFA plus the multidomain intervention 3) the multidomain intervention plus placebo,<br>4) placebo alone.	5 (High)

8	Jun el. (2016)	double-blind RCT	PUFA<br>corn oil (Con)	4 (Good)
9	Claire. el. (2021)	single center, parallel, double-blind, RCT	Electrical Muscle Stimulation Procedures+<br>1)placebo capsules<br>2)Whey protein isolate capsules<br>3)WPI+Omega-4+rutin, and curcumin	4 (Good)
10	Gordon. el. (2015)	double-blind RCT	Corn oil (CON)<br>n–3 PUFA group	3 (Medium)
11	Gordon. el. (2011)	RCT	omega-3 fatty acids (Exp)<br>corn oil(Con)	4 (Good)
12	Stephen. el. (2009)	double-blind, RCT	alpha-linolenic acid (ALA)<br>placebo (Con)	4 (Good)
13	Mats el. (2020)	double-blind, RCT	Resistance exercise and omega-3 (Exp)<br>placebo (Con)	5 (High)
